# Seroconversion and seroreversion rates of anti-*Strongyloides* IgG in rural areas of the Amazon: a population-based panel study

**DOI:** 10.1590/S1678-9946202466072

**Published:** 2024-12-06

**Authors:** Fabiana Martins de Paula, Bruna Barroso Gomes, Dirce Mary Correia Lima Meisel, William Henry Roldan, Mônica da Silva Nunes, Marcelo Urbano Ferreira, Ronaldo Cesar Borges Gryschek

**Affiliations:** 1 Universidade de São Paulo, Faculdade de Medicina, Hospital das Clinicas, Laboratório de Investigação Médica (LIM-06), São Paulo, São Paulo, Brazil; 2 Universidade de São Paulo, Faculdade de Medicina, Instituto de Medicina Tropical de São Paulo, São Paulo, São Paulo, Brazil; 3 Universidade Federal de São Carlos, Departamento de Medicina, São Carlos, São Paulo, Brazil; 4 Universidade de São Paulo, Instituto de Ciências Biomédicas, Departamento de Parasitologia, São Paulo, São Paulo, Brazil; 5 Universidade NOVA Lisboa, Instituto de Higiene e Medicina Tropical, Saúde Global e Medicina Tropical, Lisboa, Portugal

**Keywords:** Strongyloides, Serology, Seroconversion, Seroreversion, Brazil

## Abstract

Using a panel study design, we aimed to estimate the seroconversion and seroreversion rates of anti-*Strongyloides* IgG antibodies from surveys carried out 11 months apart in a rural community in the Amazon Basin in Brazil. We used enzyme immunoassays to measure anti-*Strongyloides* IgG antibodies in 325 baseline plasma samples and 224 others that were collected 11 months later from residents in the agricultural settlement of Granada, Acre State. We observed anti-*Strongyloides* IgG antibodies in 21.8% of the baseline samples (which showed that 3.4% of participants had larvae in their stool) and in 23.7% of the follow-up samples. The seroconversion rate estimated at 9.7 episodes/100 person-years at risk agrees with ongoing transmission. Specific antibodies were relatively short-lived and nine (25.0%) of 36 seropositive participants at baseline were seronegative when retested 11 months later. Fecal surveys can severely underestimate the prevalence of *S. stercoralis* infection in rural Amazonians. Serology provides a field-deployable diagnostic tool to find high-prevalence populations, identify associated risk factors, and monitor intervention programs.

## INTRODUCTION

*Strongyloides stercoralis* infection is one of the major soil-transmitted helminth infections worldwide, infecting an estimated 600 million people^1 2^. Most infections are asymptomatic and limited to the gastrointestinal tract. However, unlike other soil-transmitted nematodes, *S. stercoralis* can autoinfect, which can result in chronic disease lasting decades or cause overwhelming hyperinfection or disseminated disease in people taking immunosuppressive drugs or who have impaired Th2 type cell-mediated immunity, particularly those infected with the human T-lymphotropic virus 1. These conditions can be associated with fatal outcomes^2,3^.

The diagnosis of strongyloidiasis currently relies on the microscopic identification of larvae in stool samples, a method with limited sensitivity^4,5^. Studies indicate the need for more sensitive diagnostic methods for a better understanding of the distribution of strongyloidiasis in Brazil^4,6^ and other tropical and subtropical areas^7^. Several enzyme immunoassays are available to identify anti-*Strongyloides* antibodies in serum or plasma, with a > 90% sensitivity. However, cross-reactions in patients with other nematode infections may yield false-positive tests and antibody testing are unable to reliably differentiate current from past infection^2,4^.

The prevalence and geographic distribution of *S. stercoralis* infection remain scarcely studied in Brazil. Most prevalence data originate from convenience samples of urban populations of certain regions of Brazil. In total, two pooled analysis of available data could estimate the nationwide prevalence at 5.2%^8^ and 11.2%^1^. Microscopy positivity rates ranged from 0.8 to 14.1% in the Amazonas State, the only Amazonian state for which stool-based prevalence data were available^8^. Thus, Brazil lacks population-based seroprevalence data for *S. stercoralis*.

This panel study combines data from two consecutive population-based serosurveys that measured anti-*Strongyloides* IgG antibodies in rural Amazonians. The prevalence of specific antibodies and the seroconversion and seroreversion rates between surveys in residents in a farming settlement in the Amazon Basin in Brazil were estimated. Also, a brief discussion on how these findings may inform control interventions in endemic settings in the tropics will be presented.

## MATERIALS AND METHODS

### Study area

A longitudinal cohort study was set up in March 2004 to address a range of infectious diseases of public health significance in rural Amazonians^9-13^. The study site, called Ramal do Granada, belongs to the Pedro Peixoto Settlement, in the Acrelandia municipality, Acre State. With its equatorial humid climate, this area receives most rainfall (annual average, 2,198.5 mm) from December to March. Its mean annual temperature equals 24.5 °C. This is one of the largest farming settlements opened in the Amazon Basin of Brazil in the mid-1980s^10^, which is inhabited mostly by migrants from Southeastern and Southern Brazil who are engaged in subsistence and commercial agriculture, lumbering, and cattle raising. Coffee, banana, and rice are the main cash crops.

### Baseline survey

During the baseline survey in 2004, shortly after the rainy season, a total of 473 inhabitants were enumerated during a census in Granada. They were aged from one day to 90 years and were distributed into 114 households. Sociodemographic and morbidity information was collected from 467 (98.7%) consenting residents or their parents or guardians, who lived in 113 households. Venous blood samples were obtained from 438 (92.6%) participants. All age groups were systematically sampled except for children who were aged under five years, who were only bled at request of their parents or guardians. Overall, frozen aliquots of 325 baseline plasma samples (74.2% of those collected in March 2004) were available for anti-*Strongyloides* antibody testing.

Participants in the baseline survey, regardless of their age, were given plastic containers containing 10% formalin and asked to provide a stool sample; 429 (90.7%) had one specimen examined by the sedimentation-concentration method^9^. Participants harboring intestinal parasites were given standard, unsupervised treatment with mebendazole for intestinal nematodes other than *S. stercoralis*, albendazole for tapeworms, thiabendazole for *S. stercoralis*, and metronidazole for *Giardia duodenalis* or *Entamoeba histolytica/E. dispar.* The thiabendazole regimen used during the baseline study (50mg/kg/day divided every 12 h, with a maximum of 3 g/day for two days) is as efficacious as standard ivermectin (200 μg/kg/day for two days), although adverse events were less common with ivermectin^14^. No post-treament stoll samples were systematically collected and examined to document cure.

### Follow-up survey

Households were revisited 11 months after the baseline survey. Of the 394 study participants interviewed during the follow-up visit, 224 (56.8%) had a plasma sample aliquot stored at −20 °C available for anti-*Strongyloides* antibody testing. Overall, 158 participants had plasma samples collected during both surveys and comprised the population sample of the seroconversion/seroreversion study. No stool sample were obtained during follow-up survey.

### Antigen preparation

An extract of third-stage larvae (L3i) was prepared as described^15^ and used as solid-phase antigen. Briefly, approximately 200 mg (dry weight) of *Strongyloides venezuelensis* L3i. Larvae were obtained and resuspended in saline buffer at pH 7.2 containing 0.25% of the cationic detergent cetyltrimethylammonium bromide and incubated overnight at 4 °C. The suspension was centrifuged (12,400 ×*g* for 10 min) and the supernatant was collected and stored at −20 °C until use as an antibody-capture antigen.

### Antibody detection

The enzyme linked immune sorbent assay (ELISA) used in this study for IgG detection has an estimated 95.0% diagnostic sensitivity, with a 97.8% specificity and a 0.286 cut-off^15^. High-affinity 96-well microplates were incubated overnight at 4 °C with antigen diluted in carbonate/bicarbonate buffer (pH 9.6). Then, the plates were incubated with PBS-T plus 5% non-fat milk (PBS-TM). Plasma samples were added in duplicate at a 1/200 dilution in PBS-TM. Next, peroxidase-labeled anti-human IgG antibody (Sigma-Aldrich, St. Louis, MI, USA), diluted at 1/10,000 in PBS-TM, was added. The 3,3’, 5,5’-tetramethylbenzidine chromogen solution (Thermo Fischer Scientific, Waltham, MA, USA) was added for a seven-min incubation at room temperature in the dark. Finally, the reaction was stopped with 2N sulfuric acid and absorbance was measured at 450 nm using an ELISA reader. All microplates included positive controls (pool of antibody-positive plasma samples from people with *S. stercoralis* infections, as confirmed by parasitological diagnosis) and negative controls (pool of antibody-negative plasma samples from people with infection with soil-transmitted helminths ruled out by parasitological diagnosis), as well as blank controls (no plasma added to the microplate wells). All samples with absorbance values greater than the cutoff value were classified as positive.

### Statistical analysis

Data were analyzed with Stata 15.1 (StataCorp, College Station, TX, USA). Multivariable Poisson's regression models were run to identify correlates of positive *Strongyloides* serology at the baseline and follow-up surveys^16^. Given our relatively small sample size (e.g., 325 participants screened for specific antibodies at baseline) and the low prevalence of the binary outcome (approximately one-fourth of participants had detectable antibodies), stringent criteria were used to select variables to be included in multiple regression models^17^. Indeed, only variables associated with *P* <0.20 in initial unadjusted analyses were entered in multivariable regression models that were built separately for the baseline survey and both surveys combined. Covariates were introduced in multivariable models in a forward stepwise approach, and only those that were associated with the outcome at a significance level of at least 10% were retained in the final model. Adjusted prevalence ratio (PR) estimates are provided along with 95% confidence intervals (CI) to quantify the influence of each predictor on the outcome (antibody positivity) while controlling for all other covariates^16^. Infection incidence was estimated as the number of seroconverters per 100 person-years at risk and its exact Poisson's 95% confidence interval (CI) was calculated, with time at risk defined as time interval between blood draws.

### Ethical considerations

This human study protocol was analyzed and approved by the Institutional Review Board of the Instituto de Ciencias Biomedicas, Universidade de Sao Paulo (protocols Nº 318/CEP and 538/CEP). The maintenance of the *S. venezuelensis* strain was approved by the Animal Research Ethics Committee of the Faculdade de Medicina da Universidade de Sao Paulo (protocol Nº 0356A).

## RESULTS

### Baseline antibody prevalence and associated risk factors

*Strongyloides* IgG antibodies occurred in 71 of 325 participants at baseline (seroprevalence rate: 21.8%; 95% CI 17.7–26.6%) who were aged from six to 90 years (mean: 30.4 years). The prevalence of intestinal parasites in this community (including coinfections with different species) has been described elsewhere^9^. Overall, 41 (14.7%) participants had eggs or larvae of one or more soil-transmitted helminth species in their feces, and nine (81.8%) of the 11 participants passing *S. stercoralis* larvae in their stools (overall stool-based prevalence of infection: 3.4%) were IgG-positive. Unadjusted analysis identified age> 5 years, low wealth strata, and lack of flush toilet in the household to be significantly associated with baseline seropositivity (Table 1). Infections with *Trichuris trichiura* and hookworm diagnosed by baseline stool examination, but not those with *Ascaris lumbricoides* and the intestinal protozoon *Giardia duodenalis*, were also associated with a significantly increased *Strongyloides* seropositivity rate. Antibody positivity showed no significant association with recent wheezing or anemia (Table 1).

**Table 1:** Factors associated with baseline IgG antibody positivity to *Strongyloides stercoralis* in rural Amazonians (n = 325) according to unadjusted analysis.

Parameter		Nº tested	% seropositive	PR	95% CI	*P*
**Individual characteristic**						
**Age (years)**						
	≤5	9	0.0	Reference		
	6–15	101	16.8	1.17	(1.06 1.28)	0.001
	16–30	100	23.0	1.23	(1.14 1.35)	<0.0001
	31–49	74	32.4	1.23	(1.13 1.34)	<0.0001
	≥50	41	34.1	1.34	(1.20 1.50)	<0.0001
**Sex**						
	Female	157	18.5	Reference		
	Male	168	25.0	1.05	(0.98 1.13)	0.133
**Recent wheezing (< 12 months)**						
	No	281	21.0	Reference		
	Yes	38	28.9	1.06	(0.95 1.20)	0.284
**Anemia**						
	No	267	21.7	Reference		
	Yes	52	19.2	0.98	(0.88 1.09)	0.700
**Infection with one or more soil-transmitted helminths**						
	No	237	18.6	Reference		
	Yes	41	46.3	1.23	(1.08 1.40)	0.001
**Infection with *Strongyloides***						
	No	267	20.2	Reference		
	Yes	11	81.2	1.51	(1.31 1.74)	<0.0001
**Infection with *Ascaris***						
	No	262	21.0	Reference		
	Yes	16	50.0	1.24	(0.99 1.56)	0.066
**Infection with hookworm**						
	No	253	20.5	Reference		
	Yes	25	44.0	1.19	(1.06 1.34)	0.003
**Infection with *Trichuris***						
	No	270	21.5	Reference		
	Yes	8	62.5	1.34	(1.10 1.62)	0.003
**Infection with *Giardia***						
	No	227	22.9	Reference		
	Yes	51	21.6	0.99	(0.82 1.10)	0.835
**Household characteristic**						
**Wealth index quartile**						
	1^st^ (poorest)	76	35.5	Reference		
	2^nd^	92	23.9	0.91	(0.78 1.07)	0.275
	3^rd^	73	16.4	0.86	(0.74 0.99)	0.041
	4^th^ (least poor)	84	11.9	0.83	(0.72 0.95)	0.007
**Predominant housing material**						
	Bricks	12	16.7	Reference		
	Wood	313	22.0	1.05	(0.81 1.35)	0.730
**Water source**						
	River or spring	16	43.7	Reference		
	Groundwater well	309	20.7	0.84	(0.69 1.01)	0.072
**Type of latrine in the house**						
	Pit latrine or none (open defecation)	271	24.3	Reference		
	Flush toilet	53	9.4	0.88	(0.79 0.97)	0.015

PR = prevalence rate ratio; CI = confidence interval; Number of individuals differ for some variables because of missing values.

Age and infection with one or more soil-transmitted helminths (i.e., *Ascaris, Trichuris*, hookworm or *S. stercoralis*, including coinfections with different species) configured independent individual predictors of baseline *Strongyloides* IgG positivity after controlling for potential confounders. Poverty and the lack of a groundwater well and of a flush toilet in the household constituted significant household risk factors (Table 2). Next, we ran separate multivariable models to test whether *Strongyloides* IgG positivity was independently associated with a stool-based diagnosis of baseline infection with each of the most prevalent soil-transmitted helminths, namely *Ascaris, Trichuris*, hookworm, and *S. stercoralis*. We found significant and independent associations between *Strongyloides* IgG positivity and concurrent infection with hookworm (PR = 1.15, 95% 1.02–1.29, *P* = 0.019), *Trichuris trichiura* (PR = 1.35, 95% 1.11–1.64, *P* = 0.003), and *S. stercoralis* (PR = 1.44, 95% 1.24–1.66, *P* < 0.0001) but none with *Ascaris lumbricoides* (PR = 1.19, 95% 0.97–1.46, *P* = 0.096).

**Table 2:** Factors associated with baseline IgG antibody positivity to *Strongyloides stercoralis* in rural Amazonians (n = 277) according to mixed-effects multiple Poisson's regression analysis.

Parameter		Adjusted PR	95% CI	*P*
**Age**				
	≤5	Reference		
	6–15	1.17	(1.06 1.28)	0.001
	16–30	1.23	(1.14 1.35)	<0.0001
	31–49	1.23	(1.13 1.34)	<0.0001
	≥50	1.34	(1.20 1.50)	<0.0001
		*P* for trend = 0.002		
**Infection with one or more soil-transmitted helminths**				
	No	Reference		
	Yes	1.18	(1.05 1.32)	0.005
**Wealth index quartile**				
	1^st^ (poorest)	Reference		
	2^nd^	0.97	(0.83 1.13)	0.695
	3^rd^	0.90	(0.78 1.03)	0.126
	4^th^ (least poor)	0.87	(0.77 0.98)	0.025
		*P* for trend = 0.009		
**Water source**				
	River or spring	Reference		
	Groundwater well	0.77	(0.66 0.91)	0.002
**Type of latrine in the house**				
	Pit latrine or none (open defecation)	Reference		
	Flush toilet	0.90	(0.82 0.99)	0.031

PR = prevalence rate ratio; CI = confidence interval.

### Antibody prevalence at the follow-up survey and associated risk factors

We found *Strongyloides* IgG antibodies in 53 of 224 people, who were aged from seven to 63 years (mean, 31.4 years) and were examined at the follow-up survey. Their estimated seropositivity rate totaled 23.7% (95% CI 18.6–29.6%) during the second visit. We ran a mixed-effects Poisson's regression model with the complete dataset comprising 549 samples (124 of those tested positive) to identify independent predictors of IgG seropositivity among 391 individuals who participated in one or both study visits. Because the follow-up did not collect stool nor and hemoglobin samples, we were unable to examine the association of IgG seropositivity with anemia and soil-transmitted helminth infection when combining data from the baseline and follow-up visits. We confirmed age >five years and lack of a flush toilet as independent risk factors for IgG seropositivity in the study population, whereas the availability of a groundwater well and increasing wealth were associated with lower seropositivity (Table 3).

**Table 3:** Factors associated with IgG antibody positivity to *Strongyloides stercoralis* in rural Amazonians (n = 549) according to mixed-effects multiple Poisson's regression analysis; combined data from two cross-sectional surveys.

Parameter		Adjusted PR	95% CI	*P*
**Age**				
	≤5	Reference		
	6–15	1.23	(1.09 1.39)	0.001
	16–30	1.29	(1.17 1.43)	<0.0001
	31–49	1.37	(1.22 1.53)	<0.0001
	≥50	1.37	(1.20 1.55)	<0.0001
		*P* for trend = 0.002		
**Wealth index quartile**				
	1^st^ (poorest)	Reference		
	2^nd^	0.96	(0.83 1.11)	0.567
	3^rd^	0.87	(0.77 0.98)	0.022
	4^th^ (least poor)	0.84	(0.74 0.94)	0.002
		*P* for trend =<0.0001		
**Water source**				
	River or spring	Reference		
	Groundwater well	0.87	(0.76 0.99)	0.032
**Type of latrine in the house**				
	Pit latrine or none (open defecation)	Reference		
	Flush latrine	0.88	(0.81 0.96)	0.006

PR = prevalence rate ratio; CI = confidence interval.

### Changes in antibody status between surveys

**Figure 1:**
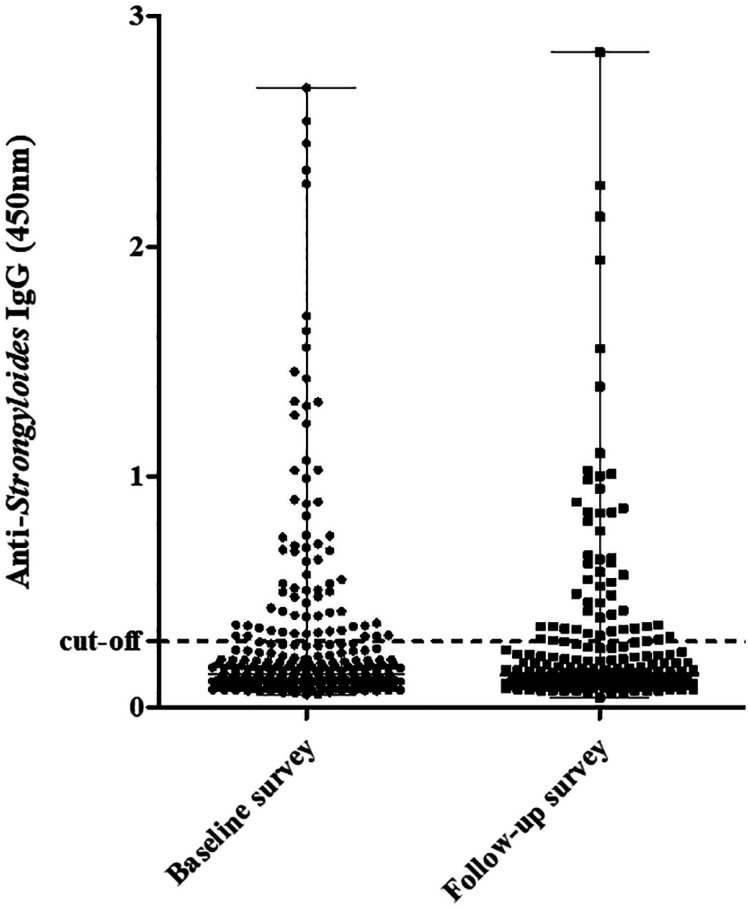
Anti-*Strongyloides* IgG (450nm) of plasma samples from Amazonians tested at baseline (n=325) and follow-up surveys (n=224). The dashed lines represent the positivity threshold (cut-off 0.286). Horizontal bar indicates the median.

Both surveys found similar absorbance values of the positive samples. Figure 1 shows the distribution of absorbance values of the baseline and follow-up surveys plasma samples by IgG-ELISA.

**Figure 2:**
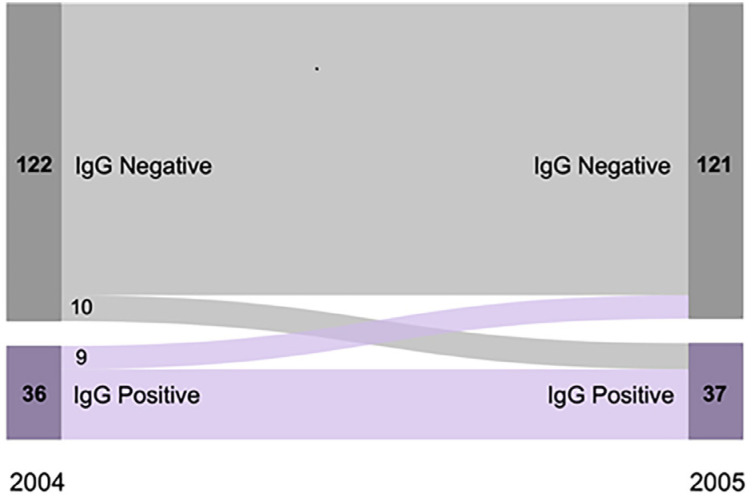
Distribution of rural Amazonians tested both at baseline and 11 months later (n = 158) according to *Strongyloides* IgG status in each serosurvey.

Overall, 19 (12.0%) of the 158 individuals tested 11 months after the baseline changed their initial antibody status (i.e., seroconverted or seroreversed) (Figure 2). The estimated seroconversion rate equaled 9.7 (95% CI 4.9–17.3) episodes/100 person-years at risk (10 seroconverters among 122 individuals who were antibody-negative at baseline and were tested in 2005), consistent with an annual incidence of *S. stercoralis* infection of nearly 10% in the community. Moreover, nine (25.0%) of the 36 individuals who tested antibody-positive at the baseline were seronegative when retested, with a seroreversion rate estimated at 27.3 (95% CI 13.3–50.0) episodes/100 person-years.

## DISCUSSION

Since strongyloidiasis is now part of the World Health Organization roadmap for neglected tropical diseases targeted for control in 2021−2030^18^, more sensitive diagnostic tools applicable to large-scale surveys are urgently needed to identify populations eligible for preventive mass chemotherapy and monitor the outcome of preventive chemotherapy and other control interventions. This population-based panel study, with repeated antibody measurements in the same community 11 months apart, shows that at least one-fifth of the examined rural Amazonians had been exposed to *S. stercoralis,* although only 3.4% of participants examined at baseline had larvae in a single stool sample.

The prevalence, incidence, and risk factors for infection with *S. stercoralis*, compared with other soil-transmitted helminths such as *Ascaris lumbricoides* and hookworm, remain scarcely studied in rural populations of the Americas^6,19-21^. This is likely due to the lack of practical and sensitive fecal-based diagnostic techniques appropriate for large-scale use in hard-to-reach populations. Indeed, most surveys rely on direct smear examination or simple sedimentation-concentration methods to identify larvae in fecal samples. More sensitive tests, such as the Baermann concentration method—which uses the hydrotropism and thermotropism of larvae—and the agar plate culture, are laborious tests, requiring fresh stool samples and have been scarcely used for population screening across the region^4^. Multiple stool samples collected over consecutive days should ideally be processed and examined because of the intermittent release of larvae in the feces^4,5^, but this is rarely feasible in population surveys. Therefore, the analysis of a single sample using a parasitological method is poorly sensitive for larval research and may have contributed to the low positivity (3.4%) for *S. stercoralis* in the fecal samples in the baseline study.

Antibody-based tests, with a reported sensitivity of 71% to 95%^22,23^, play a major role in the diagnosis of *S. stercoralis* infections at the individual and population levels^24^. However, relatively few population-based serosurveys have examined the prevalence of anti-*S. stercoralis* antibodies in Latin American populations^6,19-21^. The stool prevalence of *S. stercoralis* infections in our study population^9^ was six-times lower than seroprevalence; likewise, stool-based prevalence estimates of *S. stercoralis* infection (with a single stool sample examined for two-thirds of participants) were almost seven-times lower than seroprevalence estimates in a recent meta-analysis^18^. We found seroprevalence rates (21.8%–23.7%) close to figures reported for rural communities on the border between Argentina and Bolivia (19.6%)^20^, but substantially lower than that found in rural Amazonians from the Department of Loreto, Peru (72%)^19^. A recent meta-analysis estimated at 11.4% the pooled prevalence of anti-*S. stercoralis* antibodies among migrants from Latin America and the Caribbean who currently live in low-endemicity countries^18^.

A major limitation of serology is its likely overestimation of *S. stercoralis* infection prevalence due to false-positive results originating from past infections or cross-reactivity with other locally circulating nematodes^7^. Despite the high specificity of our ELISA^15^, we found a statistically significant association between *Strongyloides* seropositivity and microscopy-confirmed infection with hookworm and *T. trichiura*. Notably, this association may be at least in part due to the presence of cross-reactive antibodies. Importantly, cross-reactivity goes beyond immunoassays using crude larval extracts as solid-phase antigens, such as ours. Indeed, the widely used recombinant *S. stercoralis* protein NIE shares 12–18% amino acid sequence identity with proteins from other nematodes^25^ and seems to be cross-recognized by antibodies from *Strongyloides*-uninfected individuals exposed to other helminths^7^. Likewise, false-positive results show no association with the use of homologous (*S. stercoralis*) vs. heterologous (*S. ratti* or *S. venezuelensis*) crude larval antigens for the serodiagnosis of human strongyloidiasis^26^.

Based on paired sample analysis, we estimated the community-wide annual incidence of *S. stercoralis* infection at approximately 10% and found that over one-fourth of the seropositive individuals at baseline became seronegative within 11 months. Limited research has examined seroconversion rates in people naturally exposed to S. stercoralis infections. Importantly, the time required for seroconversion remains undetermined in humans. In an Aboriginal community in Australia, 157 seronegative participants at baseline were retested for anti-S. stercoralis antibodies six months later and only four (2.5%) of them had seroconverted^27^. However, the incidence of new infections was surely affected by a community-wide intervention: study participants were treated with single-dose ivermectin at baseline, regardless of any laboratory evidence of infection.

Because of the increasing use of serology in the initial diagnosis and follow-up of strongyloidiasis in clinical settings^24^, the duration of specific antibody responses following treatment has attracted great attention in recent years. Specific antibody levels drastically decrease and often become undetectable within six months after treatment^28^, antibody-positive individuals are likely to have ongoing or relatively recent infections. Overall, we observed that the absorbance values in the two surveys were similar, which may indicate similar antigenic exposure.

Most antibody-positive patients will serorevert following effective anthelminthic therapy and patterns of post-treatment antibody titer decay have been described for travelers or migrants who are not exposed to reinfection^20^. However, seroreversion rates seem to critically depend on baseline antibody titers in heavily exposed populations. For example, two-thirds of participants in the Australian mass drug administration study who remained seropositive at month six despite ivermectin administration had high levels of antibodies at baseline^27^. These findings replicate the association between high baseline antibody titers and failure to serorevert following treatment described by Kobayashi *et al*.^29^ two decades earlier. Although our seroreversion results evade direct comparisons with those from clinical settings with extensive post-treatment laboratory follow-up, they suggest that sequential antibody measurements can be useful to monitor temporal trends and identify recent or ongoing *S. stercoralis* transmission, although optimal sampling intervals remain to be determined.

A major limitation of this study is its use of 20 year-old frozen plasma samples for antibody testing. Another limitation refers to the parasitological diagnosis, which has been limited to the baseline study, used a technique with low sensitivity for *Strongyloides* larvae detection, and the analysis of a single stool sample. However, this is the first study of seroconversion and seroreversion rates of *S. stercoralis* antibodies carried out in Brazil.

## CONCLUSION

We show that community-wide surveys relying on stool examination methods can severely underestimate prevalence rates of *S. stercoralis* infections. Furthermore, we provide evidence for the use of serology as a field-deployable diagnostic method, particularly to monitor community-wide intervention programs.
